# Factors Associated with Pain, Disability, and Quality of Life in Adults Aged 18–65 with Nonspecific Chronic Neck Pain: A Single-Center Observational Study

**DOI:** 10.3390/healthcare14081030

**Published:** 2026-04-14

**Authors:** Nerea de Miguel-Hernando, Daniel Pecos-Martín, Rubén Cámara-Calmaestra, Daniel Rodríguez-Almagro, Agustín Aibar-Almazán, Samuel Fernández-Carnero, Alfonso Javier Ibáñez-Vera, Alexander Achalandabaso-Ochoa

**Affiliations:** 1 Department of Surgery, Ophthalmology, Otorhinolaryngology and Physiotherapy, Faculty of Health Sciences, University of Valladolid, Universidad St., n/n, 42004 Soria, Spain; nerea.miguel@uva.es; 2 Department of Nursing and Physiotherapy, University of Alcalá, San Diego Sq., n/n, 28801 Alcalá de Henares, Spain; daniel.pecos@uah.es (D.P.-M.); samuel.fernandezc@uah.es (S.F.-C.); 3 Department of Health Sciences, University of Jaén, Campus Las Lagunillas n/n, 23071 Jaén, Spain; rubencamcal93@gmail.com (R.C.-C.); aaibar@ujaen.es (A.A.-A.); ajibanez@ujaen.es (A.J.I.-V.); aaochoa@ujaen.es (A.A.-O.); 4 Department of Nursing, Physical Therapy and Medicine, University of Almería, Sacramento Rd., n/n, La Cañada de San Urbano, 04120 Almería, Spain

**Keywords:** chronic pain, neck pain, disability, quality of life, cervical range of motion, drug consumption

## Abstract

**Highlights:**

**What are the main findings?**

- Female sex, reduced active cervical rotational mobility, and frequent medication use are associated with higher pain intensity, greater pain-related disability, and lower HRQoL in individuals with non-specific chronic neck pain.
- Reduced cervical rotation and higher drug consumption show significant cross-sectional associations with poorer clinical status in this population.

**What are the implications of the main findings?**

- These factors should be considered in the comprehensive clinical assessment of patients with non-specific chronic neck pain.
- Longitudinal studies are needed to determine whether these variables contribute to the development or persistence of disability and reduced HRQoL.

**Abstract:**

**Background/Objectives**: Non-specific chronic neck pain is one of the biggest problems in the current population, with high levels of pain and disability and a decrease in the quality of life. The aim of this study is to assess possible variables that may be associated with neck pain, such as disability, pain, quality of life, sex, neck muscle endurance, active range of motion (AROM) and frequency of drug use. **Methods**: We performed a cross-sectional study of non-specific chronic neck pain with a total of 105 subjects. The variables pain-related disability, pain, quality of life, sex, AROM and frequency of drug use were evaluated. **Results**: A total of 105 patients with chronic neck pain were included (mean age 40.47 ± 12.18 years; 67.6% women). Neck pain–related disability showed significant negative correlations with all cervical AROM variables, particularly left rotation (r = −0.507) and right rotation (r = −0.489) (*p* < 0.001). Disability was also negatively correlated with health-related quality of life (r = −0.604) and positively correlated with pain intensity (r = 0.414) and frequency of drug consumption (r = 0.546) (*p* < 0.001). Regression analyses indicated that disability was associated with reduced left rotation mobility and higher drug consumption (R^2^ = 0.424). Pain intensity was associated with female sex, reduced right rotation mobility, and higher drug consumption (R^2^ = 0.246). Lower health-related quality of life was associated with higher drug consumption and female sex (R^2^ = 0.174). **Conclusions**: Being female, having a reduction in active rotational mobility, and a high frequency of drug consumption are associated with greater pain-related disability and pain intensity, and a lower HRQoL in subjects with non-specific chronic neck pain.

## 1. Introduction

The term non-specific chronic neck pain is defined as a musculoskeletal disorder whose symptoms present a fluctuating course with relapsing-remitting episodes of pain of minimal duration of 3 months [1]. Neck pain is classified according to its etiology as specific pain or non-specific, with non-specific neck pain being more frequent, since up to 90% of neck pain is of unknown cause [2]. This condition is highly prevalent worldwide and represents a substantial public health concern, as neck pain is among the leading causes of years lived with disability (YLDs). In 2021, the global age-standardized rate of YLDs specifically attributable to neck pain was estimated at 242 per 100,000 population. Its global prevalence reached 203 million cases in 2020, with higher rates consistently observed in females and an age gradient that peaks between 45 and 74 years [3].

Chronic neck pain has been associated with high levels of pain and disability [3] and with a decrease in the quality of life [4], which has made it one of the entities with the highest demand for treatment in current clinical practice [5]. Therefore, in the last 5 years research on this entity has grown.

While previous literature has extensively documented the presence of pain, disability, and reduced quality of life in subjects with non-specific chronic neck pain, their simultaneous association with multiple clinical and demographic factors remains less explored. The population of interest in the present study consisted of adults aged 18 to 65 years with non-specific chronic neck pain who were seeking care at the Recoletas Burgos Hospital’s Rehabilitation Services. Consequently, the aim of this study was to assess the simultaneous association of multiple variables—such as sex, neck muscle endurance, AROM, and frequency of drug use, with pain, disability, and quality of life within a multivariate model in this specific clinical population.

## 2. Materials and Methods

### 2.1. Study Design

A cross-sectional study was conducted following the Strengthening the Reporting of Observational Studies in Epidemiology (STROBE) recommendations [6] (see Supplementary Material S1 for the completed STROBE checklist) at the Recoletas Burgos Hospital’s Rehabilitation Services on December 2019, January 2020 and September and October 2021. The study was in accordance with the Declaration of Helsinki and was approved by the aforementioned Hospital and the Ethics Committee for Research and Animal Experimentation of the University of Alcalá, which is authorized to review and approve human research protocols (CEIM/HU/2019/28).

### 2.2. Subjects

In December 2019, January 2020 and September and October 2021 consecutive participants affected by non-specific neck pain were recruited from Recoletas Burgos Hospital’s Rehabilitation Services. All participants involved read the Participant Information Sheet and signed a written informed consent.

The inclusion criteria were, suffered from non-specific neck pain for at least 3 months [7], been aged from 18 to 65 years. Participants were excluded if they presented any of the following: previous cervical surgery or whiplash injury; having received physical therapy in the 3 months prior to the study; neck pain accompanied by vertigo caused by vertebrobasilar insufficiency; or pain accompanied by non-cervicogenic headaches. Importantly, concurrent cervicogenic headache was not an exclusion criterion, as it frequently co-occurs with chronic neck pain. Additional exclusion criteria included: diagnosis of cervical radiculopathy and/or externalized herniated disk; “red flags” (such as fracture, tumor, cervical infection, osteoporosis, metabolic diseases, or rheumatoid arthritis); myopathy, ankylosing spondylitis, or fibromyalgia; central nervous system involvement or neurological symptoms; pain in other parts of the body preventing the performance of the study assessments; pregnancy; severe psychiatric or psychological disorders; pending legal action; and insufficient knowledge to understand, write, and speak Spanish fluently.

**Figure 1:**
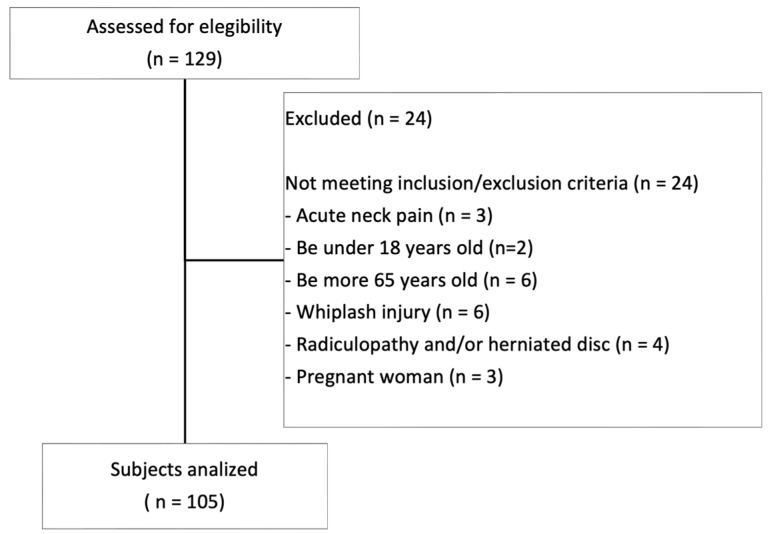
Flow diagram of subjects.

Finally, 105 participants met the eligibility criteria. The selection process is shown in Figure 1.

### 2.3. Sample Size Calculation

The sample size was justified based on Green’s regression heuristic [8] (*N* ≥ 50 + 8*k*), where “*k*” is the number of predictors. For a model including seven potential predictors, a minimum sample size of 106 participants is recommended. The final sample of 105 participants closely approximates this recommendation. Furthermore, the final regression models retained only two to three predictors, resulting in a favorable observations-to-predictor ratio and supporting the stability of the regression estimates.

### 2.4. Measurements

Demographic data (height, weight, sex and age) were collected at the beginning of the measuring session.

The pain-related disability was assessed by the Spanish version of the Neck Disability Index (NDI) [9]. This scale was shown to have good internal consistency (Cronbach’s α = 0.89) and good test–retest reliability (ICC = 0.88) in subjects with chronic non-specific neck pain [9].

Neck pain was assessed by the Numeric Pain Rating Scale (NPRS) with a score from 0 to 10 [10]. As confirmed by a recent systematic review [11], the NPRS demonstrates good to excellent test–retest reliability (ICC = 0.58–0.93) and strong concurrent validity in populations with neck pain.

Health-Related Quality of Life (HRQoL) was measured using the Spanish Version of the Short Form 36-item Health Survey [12]. This questionnaire presented a good internal consistency in all dimensions (Cronbach’s α = 0.71–0.94) except for the social function (SF) dimension (Cronbach’s α = 0.45). The test–retest reliability of this questionnaire was moderate to excellent (ICC = 0.58–0.99) [12].

Neck muscle endurance was measured using the modified Neck Extensor muscle Endurance test (modified NEE test) proposed by Lee et al. (2005) [13] and the Neck Flexor Muscle Endurance test (NFME test) [14] with a chronometer and a Baseline Digital Inclinometer (BDI). According to a recent meta-analysis [15], the NFME test demonstrated good intrarater (ICC = 0.86) and moderate interrater reliability (ICC = 0.72), while the modified NEE test showed good intrarater (ICC = 0.84) and interrater reliability (ICC = 0.81) in subjects with neck pain. Furthermore, these tests presented a standard error of measurement (SEM) of 6.91 s for the NFME and 0.74 min for the modified NEE test [14], although the test values differed according to sex [16].

Cervical spine’s Active Range of Motion was measured in flexion, extension, lateral tilt, and rotation movements using a BDI [17]. The range of motion (ROM) of the cervical spine presented good to excellent test–retest reliability (ICC = 0.80–0.94), with a highly precise SEM ranging from 1.6° to 2.6°. Furthermore, this instrument has demonstrated strong discriminative validity in subjects with chronic cervical pain [18].

Analgesic drug consumption was assessed by a Medication Record Sheet (0–7 days/week). Frequency (0–7 days/week) was used as a proxy for medication dependence and symptom persistence, which are recognized predictors of disability in chronic pain.

### 2.5. Statistical Analysis

IBM SPSS Statistics, Version 30.0 (IBM Corp., Armonk, NY, USA) was employed to data managing. The level of statistical significance was established at *p* < 0.05. Descriptive statistics, including means and standard deviations, were calculated for all continuous variables, while frequencies were used for categorical data. The normality of the distribution was assessed using the Kolmogorov–Smirnov test.

A sensitivity analysis comparing pre- and post-pandemic cohorts was performed to assess potential sample bias, as data collection was interrupted by a 20-month gap (December 2019–October 2021) due to the COVID-19 pandemic. Continuous variables were tested using Student’s *t*-test, whereas categorical variables were analyzed with the chi-square test.

First, a bivariate correlation analysis was conducted using Pearson’s correlation coefficient (r) to explore the strength and direction of the relationships between dependent variables (neck pain intensity, disability and health-related quality of life) and independent variables (AROM variables, neck muscles resistance and frequency of drug consumption).

To allow for a direct comparison of the relative weight of each predictor and to establish the strength of the associations independently of the scales of measurement, all continuous variables were standardized (transformed into Z-scores) prior to the regression analysis. Consequently, results are reported as standardized regression coefficients (St. β). This approach allowed for a direct comparison of the relative weight of each predictor, regardless of their original units of measurement.

Subsequently, univariate linear regression analyses were performed to examine the association between each dependent variable and the independent variables. Variables showing statistical significance (*p* < 0.05) in the univariate analyses were entered into multiple linear regression models.

A forward stepwise selection procedure was used as an exploratory modeling strategy to identify the variables independently associated with each outcome and to derive parsimonious regression models. Multicollinearity among predictors was assessed using variance inflation factors (VIF) and tolerance statistics. The assumptions of linear regression were evaluated by inspecting standardized residuals, normal probability plots, and scatterplots of standardized residuals versus predicted values to assess normality, linearity, and homoscedasticity.

The explanatory capacity of the multiple regression models was assessed using the coefficient of determination (R^2^). According to Cohen’s criteria [19], R^2^ values below 0.02 would be considered insignificant effect sizes, between 0.02 and 0.15 would be considered small, between 0.15 and 0.35 would be considered medium, and above 0.35 would be considered large. The statistical significance level was set at *p*-value <0.05.

## 3. Results

A total of 105 subjects (Figure 1) with chronic neck pain, with a mean age of 40.47 years (SD = 12.18) were evaluated for this study. The female population represented more than two-thirds of the total sample. On average, the participants exhibited moderate pain intensity and mild disability levels. Table 1 reflects morphological and clinical data of the sample. The Kolmogorov–Smirnov test confirmed that all main variables followed a normal distribution (*p* > 0.05). Levene’s test yielded non-significant results (*p* > 0.05), justifying the use of parametric statistics and linear regression models.

The sensitivity analysis comparing pre- and post-pandemic cohorts to evaluate potential selection bias revealed no significant differences in the clinical characteristics of the study population (Table 1).

The bivariate relationships between pain, disability, quality of life, and the independent variables are shown in Table 2.

Neck pain-related disability demonstrated significant negative correlations with all cervical AROM variables. Specifically, the strongest associations were found with left rotation (r = −0.507, *p* < 0.001) and right rotation (r = −0.489, *p* < 0.001), followed by extension (r = −0.451, *p* < 0.001) and flexion (r = −0.334, *p* < 0.001) (Table 2). These findings indicate that lower cervical mobility is consistently associated with higher levels of neck disability.

Additionally, neck pain-related disability showed a strong negative correlation with health-related quality of life (r = −0.604, *p* < 0.001) and a strong positive correlation not only with pain intensity (r = 0.414, *p* < 0.001), but also with frequency of drug (r = 0.546, *p* < 0.001) (Table 2).

Significant correlations were also observed among the independent variables. Cervical rotation (right and left) showed a very strong association (r = 0.816, *p* < 0.001), while frequency of drug was negatively associated with all AROM variables, most notably with extension (r = −0.404, *p* < 0.001) (Table 2).

The multiple linear regression analysis revealed several significant predictors for the studied variables. Standardized β coefficients were used to determine the relative contribution of each variable

Regarding neck pain-related disability, a significant model was obtained. The multiple linear regression performed showed large effect size, explaining the 42.4% of the variance of neck pain-related disability in patients with chronic neck pain (R^2^ = 0.424; *p* < 0.001). Specifically, a negative association was found between neck pain related-disability and active left rotation mobility (St. β = −0.397; *p* < 0.001), indicating that lower range of motion in left rotation is associated with higher levels of disability. Furthermore, the frequency of drug consumption showed a positive association with neck pain-related disability (St. β = 0.413; *p* < 0.001) (Table 3). No evidence of multicollinearity was observed among the predictors included in the models (VIF range: 1.12–3.03/Tolerance range: 0.33–0.89). Inspection of residual plots confirmed that the assumptions of linear regression were adequately met.

Concerning neck pain intensity, a significant model was also obtained. The multiple linear regression performed showed medium effect size, explaining the 24.6% of the variance of neck pain intensity in patients with chronic neck pain (R^2^ = 0.246; *p* < 0.001). Specifically, a negative association was found between sex and neck pain (St. β = −0.374; *p* = 0.016). Considering sex codification (male = 1; female = 0), female sex is associated with higher pain levels. In addition, right rotation active mobility (St. β = 0.184; *p* = 0.036) and frequency of drug consumption (St. β = 0.171; *p* < 0.001), showed a positive association with neck pain intensity (Table 4). No evidence of multicollinearity was observed among the predictors included in the models (VIF range: 1.01–3.01/Tolerance range: 0.33–0.99). Inspection of residual plots confirmed that the assumptions of linear regression were adequately met.

Relating to quality of life, a significant model was obtained too. The multiple linear regression performed showed a medium effect size, explaining the 17.4% of the variance of health-related quality of life in patients with chronic neck pain (R^2^ = 0.174; *p* < 0.001). Specifically, a negative association was found between drug consumption and health-related quality of life (St. β = −0.337; *p* < 0.001), indicating that higher frequency of drug consumption is associated with worse level of quality of life. Moreover, sex was positively associated with health-related quality of life (St. β = 0.256; *p* = 0.006) (Table 5). Given the coding of the variable (male = 1; female = 0), male sex was associated with higher SF-36 scores, whereas female sex was associated with poorer health-related quality of life. No evidence of multicollinearity was observed among the predictors included in the models (VIF range: 1.00–1.20/Tolerance range: 0.33–0.84). Inspection of residual plots confirmed that the assumptions of linear regression were adequately met.

## 4. Discussion

The results of this study suggest that subjects with non-specific chronic neck pain tend to experience greater intensity of neck pain if they are women, have limited AROM in rotational movement, and have a high frequency of drug use. Along the same lines, subjects with non-specific chronic neck pain have greater pain-related disability if they present limitation of active rotation and a high frequency of drug use. On the contrary, there is a negative correlation between HRQoL and the independent variables, thus subjects with non-specific chronic neck pain tend to present a lower HRQoL if they are women and have a high frequency of drug use.

Regarding sex, our findings align with a large body of literature identifying the female sex as a well-established risk factor for chronic pain, greater pain intensity, higher disability, and lower HRQoL [20,21,22]. Previous studies have proposed various mechanisms to explain these disparities, including biological factors (such as sexual dimorphisms in microglia and neuroimmune signaling) [21,23] psychological differences (such as varying pain coping strategies [24,25] and sociocultural influences. However, given the cross-sectional design of our study and the absence of biological or psychological mediator variables, our data cannot determine the underlying mechanisms of these findings. We must explicitly acknowledge that the sex-related associations observed in this study cannot distinguish between biological, psychological, or social explanations. Future longitudinal research incorporating specific biomarkers and psychological assessments is required to clarify the relative contribution of these factors to sex differences in chronic pain.

Nonspecific chronic neck pain, apart from being related to disability and pain, has also been associated with reduced cervical mobility [26,27], mainly in active rotation movements [28], a finding that is also supported by the results of the present study. Cervical rotation involves more complex physiological coupled movements due to the anatomy of the region [29,30]. In fact, most of the rotational movement of the cervical spine occurs at the upper cervical level, particularly in the C1–C2 segment [31], which may explain why dysfunction at this level could significantly affect overall rotational mobility, as compensation by the remaining segments is anatomically limited. In our multivariate analysis, an apparent laterality was observed, with reduced left rotation emerging as an independent predictor of disability, whereas reduced right rotation was associated with pain intensity. However, this difference should be interpreted with caution. In the bivariate analysis, both right and left rotation were significantly correlated with both outcomes, suggesting that cervical rotational mobility in general is related to neck pain severity and functional limitation. Moreover, both rotational variables showed a very strong inter-correlation (r = 0.816), indicating that they share a large proportion of biomechanical information. When highly correlated predictors are included simultaneously in multivariable models, they may compete to explain overlapping variance in the outcome, and variable selection procedures such as forward stepwise regression tend to retain only the predictor providing the greatest unique contribution to the model. Therefore, the laterality observed in the final models likely reflects a statistical selection effect rather than a true side-specific biomechanical mechanism. From a clinical perspective, these findings suggest that global limitations in cervical rotation, rather than deficits in a specific direction, may be more relevant to neck pain-related outcomes. Nevertheless, while this biomechanical interpretation is plausible, it remains speculative. Given the cross-sectional design of the present study, it is not possible to establish the directionality of this relationship. Consequently, it cannot be determined whether reduced cervical rotation contributes to the development of pain and disability or whether it represents a protective limitation of movement adopted by patients in response to preexisting pain.

Although neck muscle endurance was initially hypothesized to be a relevant factor, it did not emerge as a significant predictor in the multivariate models for pain intensity, disability, or HRQoL. This lack of independent association may be explained by the multifactorial and biopsychosocial nature of non-specific chronic neck pain [32]. Furthermore, the high inter-individual variability observed in our endurance measurements (SD = 52.24) suggests a highly heterogeneous functional status among participants, which likely reduced the statistical power to identify a consistent independent association in the regression models. While physical impairments such as reduced endurance are common in this population, self-reported disability and quality of life are often more strongly driven by the severity of the pain itself and the restriction of AROM [26,27]. In a multivariate model, these dominant variables likely absorb the statistical variance, rendering isolated physical capacities like muscle endurance non-significant. Furthermore, patients with chronic neck pain frequently develop compensatory motor control strategies [26,27]; thus, they may present poor endurance on clinical tests but still manage to perform daily activities, uncoupling this physical deficit from their perceived disability and overall quality of life.

Our results showed a relationship between the frequency of drug use and the intensity of pain, disability and HRQoL. However, these findings must be interpreted with caution. Given the cross-sectional design of this study, the directionality of this relationship remains ambiguous. Rather than drug consumption independently contributing to worse clinical outcomes, this association is likely influenced by reverse causality and confounding by indication. Specifically, patients experiencing more severe pain and greater disability naturally require and consume medication more frequently [33,34]. Therefore, in our models, the frequency of medication use acts primarily as a proxy or indicator for the underlying severity of the patient’s condition [35], rather than a direct cause of poorer health-related quality of life. Current CPGs support the effectiveness of non-invasive, non-pharmacological treatments in chronic pain. For chronic neck pain, manual therapy, physical therapy, acupuncture, exercise, and manipulative therapy have all been shown to be effective [20,36,37]. Physical activity has been shown to reduce disability and pain intensity in subjects with chronic spinal pain [20,38] and, with education in the neuroscience of pain, improves the quality of life [38]. Therefore, current evidence suggests a multidisciplinary and person-centered approach that encompasses physiotherapy, psychological treatment, pain education and, if necessary, pharmacological treatment [33,37].

This study has several limitations that should be considered when interpreting the findings.

First, several potentially relevant confounding variables were not included in the analysis. Important modifiable risk factors such as smoking status, alcohol consumption, nutrition, obesity, socioeconomic status, job position, and physical activity level were not assessed [20,39]. In addition, psychosocial factors commonly associated with chronic pain, including stress, perceived injustice, and sleep quality [40,41,42], were not evaluated. Non-modifiable variables such as age, which is a recognized risk factor for chronic pain and may interact with sex differences in pain prevalence [20,39,43,44], were also not incorporated into the models. The omission of these variables may partly explain the relatively modest explanatory capacity observed in some models, particularly for health-related quality of life (R^2^ = 0.174), and limits the clinical applicability of the regression analyses.

Second, limitations related to measurement should be acknowledged. Drug consumption was assessed using a Medication Record Sheet that quantified the frequency of use (0–7 days/week), which provides only a crude estimate of medication exposure. This measure does not capture the type of medication (e.g., NSAIDs, opioids, muscle relaxants), dosage, duration of treatment, or appropriateness of prescription. Consequently, the clinical interpretation of the observed association between medication frequency and the outcomes is limited. Similarly, health-related quality of life was analyzed using the total SF-36 score. Because the SF-36 is a multidimensional instrument, analyzing only the global score may obscure potentially relevant information at the domain level (e.g., physical functioning, bodily pain, or mental health).

Third, methodological aspects of the study design must be considered. The cross-sectional design precludes establishing causal relationships between cervical mobility, medication use, pain intensity, disability, and quality of life. Therefore, the associations observed in this study should be interpreted as relationships rather than cause–effect links. In particular, reverse causality is a potential concern, especially regarding drug consumption frequency, which may reflect the severity of underlying pain rather than being a determinant of worse outcomes.

Fourth, statistical considerations should also be acknowledged. Although the stepwise variable selection procedure was used as an exploratory strategy to derive parsimonious models, this approach may produce models that are sensitive to sample-specific variations and may therefore be unstable across different populations. Consequently, the regression findings should be interpreted cautiously and considered primarily hypothesis-generating.

Finally, issues related to sample characteristics and contextual factors may affect the generalizability of the findings. A potential language bias should be acknowledged, as individuals with insufficient knowledge of Spanish were excluded due to the absence of professional interpreters and the use of questionnaires validated only in Spanish. This may have reduced the representativeness of the sample by excluding certain immigrant populations. In addition, data collection was interrupted for approximately 20 months due to the COVID-19 pandemic. Although sensitivity analyses showed no significant differences between participants recruited before and after the pandemic interruption, pandemic-related changes in lifestyle, healthcare access, or medication use cannot be completely ruled out. Furthermore, participants in the present study generally presented with moderate pain intensity and mild disability; therefore, the findings may not be directly generalizable to populations with more severe clinical presentations.

Future studies using longitudinal designs, larger samples, and more comprehensive assessment of biological, psychosocial, and lifestyle factors are needed to confirm these findings and to better clarify the mechanisms underlying chronic nonspecific neck pain.

## 5. Conclusions

In conclusion, the results of this study suggest that female sex, having a reduction in active rotational mobility, and a high frequency of drug consumption are related to greater pain-related disability and pain intensity, and a lower HRQoL in subjects with non-specific chronic neck pain.

## Figures and Tables

**Table 1:** Characteristics of the subjects.

		Total Sample		Pre-PAN		Post-PAN		SA
Categorical		F	%	F	%	F	%	*p*
Sex	Female	71.00	67.6	36	66.7	35	68.6	0.830
	Male	34.00	32.4	18	33.3	16	31.4	
Continuous		Mean	SD	Mean	SD	Mean	SD	*p*
Age (years)		40.47	12.18	39.11	11.40	41.90	12.91	0.242
Height (m)		1.68	0.09	1.68	0.09	1.67	0.09	0.801
Weight (kg)		69.30	13.68	69.6	13.37	69.00	14.13	0.825
BMI (kg/m^2^)		24.58	4.16	24.65	4.26	24.51	4.08	0.872
Pain (NPRS, 0–10)		5.64	1.80	5.41	1.67	5.88	1.92	0.178
Disability (NDI, 0–50)		9.63	5.95	10.43	5.81	8.80	6.04	0.164
Quality of life (SF-36, 0–100)		68.89	14.29	68.63	14.61	69.15	14.08	0.852
Flexion (°)		45.36	12.46	46.18	12.01	44.48	13.00	0.488
Extension (°)		42.82	12.81	42.31	12.30	43.37	13.44	0.675
Right Side bending (°)		31.76	8.20	31.32	8.39	32.24	8.05	0.574
Left Side bending (°)		32.16	8.18	32.46	9.04	31.84	7.22	0.704
Right Rotation (°)		57.57	16.42	56.63	16.27	55.84	16.62	0.807
Left Rotation (°)		56.25	16.36	58.44	17.27	56.63	15.58	0.578
Neck Muscle Resistance (s)		61.22	52.24	59.38	51.05	63.16	53.92	0.714
Drugs Frequency (days/week)		1.29	2.02	0.96	1.75	1.64	2.25	0.088

Abbreviations: F: Frequency; %: percentage; SD: standard deviation; BMI: Body mass index. SA: sensitivity analysis; *p*: *p*-value; NPRS: Numeric Pain Rating Scale; NDI: Neck Disability Index; SF-36: Spanish Version of the Short Form 36-item Health Survey; °: degree; s: second.

**Table 2:** Correlation matrix between study variables.

Variables	1	2	3	4	5	6	7	8	9	10	11	12
1	-	-	-	-	-	-	-	-	-	-	-	-
2	0.414 **	-	-	-	-	-	-	-	-	-	-	-
3	−0.349 **	−0.604 **	-	-	-	-	-	-	-	-	-	-
4	−0.246 *	−0.115	0.270 **	-	-	-	-	-	-	-	-	-
5	−0.241 *	−0.334 **	0.162	0.086	-	-	-	-	-	-	-	-
6	−0.293 **	−0.451 **	0.215 *	0.059	0.532 **	-	-	-	-	-	-	-
7	−0.164	−0.346 **	0.088	0.100	0.434 **	0.506 **	-	-	-	-	-	-
8	−0.174	−0.293 **	0.118	0.039	0.471 **	0.458 **	0.709 **	-	-	-	-	-
9	−0.335 **	−0.489 **	0.287 **	0.079	0.389 **	0.616 **	0.637 **	0.561 **	-	-	-	-
10	−0.294 **	−0.507 **	0.265 **	0.063	0.450 **	0.516 **	0.588 **	0.639 **	0.816 **	-	-	-
11	−0.240 *	−0.180	0.105	0.141	0.330 **	0.219 *	0.089	0.123	0.222 *	0.263 **	-	-
12	0.435 **	0.546 **	−0.357 **	−0.059	−0.327 **	−0.404 **	−0.229 *	−0.241 *	−0.336 **	−0.328 **	−0.180	-

Abbreviations: 1: Pain; 2: Disability; 3: Quality of Life; 4: Sex; 5: Flexion; 6: Extension; 7: Right Side bending; 8: Left Side bending; 9: Right Rotation; 10: Left Rotation; 11: Neck Muscle Resistance; 12: Drugs Frequency; *: *p* < 0.05; **: *p* < 0.001.

**Table 3:** Univariate and multivariate linear regression to analyze the factors associated with neck pain-related disability.

Variable	Univariate Analysis				Multivariate Analysis			
	St. β	95% C.I.		*p*	St. β	95% C.I.		*p*
		Lower	Upper			Lower	Upper	
Sex	−0.115	−0.663	0.171	0.245	-	-	-	-
Flexion	−0.334	−0.517	−0.149	<0.001	−0.052	−0.243	0.140	0.593
Extension	−0.451	−0.628	−0.275	<0.001	−0.069	−0.289	0.150	0.533
Right Side bending	−0.346	−0.531	−0.160	<0.001	−0.051	−0.291	0.189	0.675
Left Side bending	−0.293	−0.480	−0.103	0.003	0.156	−0.081	0.391	0.196
Right Rotation	−0.489	−0.661	−0.316	<0.001	−0.077	−0.376	0.223	0.611
Left Rotation	−0.507	−0.675	−0.336	<0.001	−0.397	−0.557	−0.239	<0.001
Neck Muscle Resistance	−0.180	−0.372	0.013	0.067	-	-	-	-
Drugs Frequency	0.546	0.378	0.707	<0.001	0.413	0.252	0.567	<0.001

Abbreviations: St. β: standardized regression beta coefficient; 95% C.I.: 95% Confidence Interval; *p*: *p*-value.

**Table 4:** Univariate and multivariate linear regression to analyze the factors associated with neck pain intensity.

Variable	Univariate Analysis				Multivariate Analysis			
	St. β	95% C.I.		*p*	St. β	95% C.I.		*p*
		Lower	Upper			Lower	Upper	
Sex	−0.246	−0.926	−0.121	0.011	−0.211	−0.374	−0.013	0.016
Flexion	−0.241	−0.430	−0.051	0.013	0.002	−0.216	0.219	0.989
Extension	−0.293	−0.481	−0.105	0.003	−0.041	−0.290	0.208	0.744
Right Side bending	−0.164	−0.358	0.030	0.097	-	-	-	-
Left Side bending	−0.174	−0.367	0.020	0.078	-	-	-	-
Right Rotation	−0.335	−0.521	−0.150	<0.001	−0.184	−0.509	−0.143	0.036
Left Rotation	−0.294	−0.483	−0.107	0.002	0.039	−0.271	0.350	0.803
Neck Muscle Resistance	−0.240	−0.430	−0.050	0.014	−0.112	−0.296	0.163	0.233
Drugs Frequency	0.435	0.257	0.609	<0.001	0.33	0.171	0.531	<0.001

Abbreviations: St. β: standardized regression beta coefficient; 95% C.I.: 95% Confidence Interval; *p*: *p*-value.

**Table 5:** Univariate and multivariate linear regression to analyze the factors associated with health-related quality of life.

Variable	Univariate Analysis				Multivariate Analysis			
	St. β	95% C.I.		*p*	St. β	95% C.I.		*p*
		Lower	Upper			Lower	Upper	
Sex	0.270	0.175	0.975	0.005	0.256	0.163	0.923	0.006
Flexion	0.162	−0.031	0.355	0.099	-	-	-	-
Extension	0.215	0.023	0.406	0.029	−0.033	−0.271	0.206	0.785
Right Side bending	0.088	−0.107	0.284	0.372	-	-	-	-
Left Side bending	0.118	−0.077	0.312	0.234	-	-	-	-
Right Rotation	0.287	0.099	0.475	0.003	0.152	−0.183	0.487	0.369
Left Rotation	0.265	0.075	0.454	0.007	0.048	−0.264	0.361	0.756
Neck Muscle Resistance	0.105	−0.089	0.299	0.286	-	-	-	-
Drugs Frequency	−0.357	−0.543	−0.175	<0.001	−0.337	−0.516	−0.158	<0.001

Abbreviations: St. β: standardized regression beta coefficient; 95% C.I.: 95% Confidence Interval; *p*: *p*-value.

## Data Availability

The data that support the findings of this study are available from the corresponding author upon reasonable request.

## Supplementary Materials

The following supporting information can be downloaded at: https://www.mdpi.com/article/10.3390/healthcare14081030/s1, S1: STROBE Statement—Checklist of items that should be included in reports of cross-sectional studies.

## Abbreviations

The following abbreviations are used in this manuscript:

NDI	Neck Disability Index
NPRS	Numerical Pain Rating Scale
HRQoL	Health-Related Quality of Life
SF	Social Function
NEE	Neck Extensor Muscle Endurance test
NFME	Neck Flexor Muscle Endurance test
BDI	Baseline Digital Inclinometer
SEM	Standard Error of Measurement
AROM	Active Range of Motion
ROM	Range of Motion

## References

[1] Treede R.-D., Rief W., Barke A., Aziz Q., Bennett M.I., Benoliel R., Cohen M., Evers S., Finnerup N.B., First M.B. (2019). Chronic Pain as a Symptom or a Disease: The IASP Classification of Chronic Pain for the International Classification of Diseases (ICD-11). Pain.

[2] Margetis K., Singh C., Casiano V.E., Varacallo M.A. (2025). Back Pain. StatPearls.

[3] (2024). GBD 2021 Neck Pain Collaborators Global, Regional, and National Burden of Neck Pain, 1990-2020, and Projections to 2050: A Systematic Analysis of the Global Burden of Disease Study 2021. Lancet Rheumatol..

[4] Castellini G., Pillastrini P., Vanti C., Bargeri S., Giagio S., Bordignon E., Fasciani F., Marzioni F., Innocenti T., Chiarotto A. (2022). Some Conservative Interventions Are More Effective than Others for People with Chronic Non-Specific Neck Pain: A Systematic Review and Network Meta-Analysis. J. Physiother..

[5] Cieza A., Causey K., Kamenov K., Hanson S.W., Chatterji S., Vos T. (2021). Global Estimates of the Need for Rehabilitation Based on the Global Burden of Disease Study 2019: A Systematic Analysis for the Global Burden of Disease Study 2019. Lancet.

[6] Cuschieri S. (2019). The STROBE Guidelines. Saudi J. Anaesth..

[7] Fredin K., Lorås H. (2017). Manual Therapy, Exercise Therapy or Combined Treatment in the Management of Adult Neck Pain—A Systematic Review and Meta-Analysis. Musculoskelet. Sci. Pract..

[8] Green S.B. (1991). How Many Subjects Does It Take To Do A Regression Analysis. Multivar. Behav. Res..

[9] Andrade Ortega J.A., Delgado Martínez A.D., Ruiz R.A. (2010). Validation of the Spanish Version of the Neck Disability Index. Spine.

[10] Hjermstad M.J., Fayers P.M., Haugen D.F., Caraceni A., Hanks G.W., Loge J.H., Fainsinger R., Aass N., Kaasa S. (2011). Studies Comparing Numerical Rating Scales, Verbal Rating Scales, and Visual Analogue Scales for Assessment of Pain Intensity in Adults: A Systematic Literature Review. J. Pain. Symptom Manag..

[11] Modarresi S., Lukacs M.J., Ghodrati M., Salim S., MacDermid J.C., Walton D.M., CATWAD Consortium Group (2021). A Systematic Review and Synthesis of Psychometric Properties of the Numeric Pain Rating Scale and the Visual Analog Scale for Use in People With Neck Pain. Clin. J. Pain..

[12] Alonso J., Prieto L., Antó J.M. (1995). The Spanish version of the SF-36 Health Survey (the SF-36 health questionnaire): An instrument for measuring clinical results. Med. Clin..

[13] Lee H., Nicholson L.L., Adams R.D. (2005). Neck Muscle Endurance, Self-Report, and Range of Motion Data from Subjects with Treated and Untreated Neck Pain. J. Manip. Physiol. Ther..

[14] Lourenço A.S., Lameiras C., Silva A.G. (2016). Neck Flexor and Extensor Muscle Endurance in Subclinical Neck Pain: Intrarater Reliability, Standard Error of Measurement, Minimal Detectable Change, and Comparison With Asymptomatic Participants in a University Student Population. J. Manip. Physiol. Ther..

[15] Selistre L.F.A., de Sousa Melo C., de Noronha M.A. (2021). Reliability and Validity of Clinical Tests for Measuring Strength or Endurance of Cervical Muscles: A Systematic Review and Meta-Analysis. Arch. Phys. Med. Rehabil..

[16] Parazza S., Vanti C., O’Reilly C., Villafañe J.H., Tricás Moreno J.M., Estébanez De Miguel E. (2014). The Relationship between Cervical Flexor Endurance, Cervical Extensor Endurance, VAS, and Disability in Subjects with Neck Pain. Chiropr. Man. Ther..

[17] Prushansky T., Deryi O., Jabarreen B. (2010). Reproducibility and Validity of Digital Inclinometry for Measuring Cervical Range of Motion in Normal Subjects. Physiother. Res. Int..

[18] Jørgensen R., Ris I., Falla D., Juul-Kristensen B. (2014). Reliability, Construct and Discriminative Validity of Clinical Testing in Subjects with and without Chronic Neck Pain. BMC Musculoskelet. Disord..

[19] Cohen J. (1992). A Power Primer. Psychol. Bull..

[20] Skelly A.C., Chou R., Dettori J.R., Turner J.A., Friedly J.L., Rundell S.D., Fu R., Brodt E.D., Wasson N., Kantner S. (2020). Noninvasive Nonpharmacological Treatment for Chronic Pain: A Systematic Review Update.

[21] Ji R.-R., Nackley A., Huh Y., Terrando N., Maixner W. (2018). Neuroinflammation and Central Sensitization in Chronic and Widespread Pain. Anesthesiology.

[22] Suso-Ribera C., Martínez-Borba V., Martín-Brufau R., Suso-Vergara S., García-Palacios A. (2019). Individual Differences and Health in Chronic Pain: Are Sex-Differences Relevant?. Health Qual. Life Outcomes.

[23] Presto P., Mazzitelli M., Junell R., Griffin Z., Neugebauer V. (2022). Sex Differences in Pain along the Neuraxis. Neuropharmacology.

[24] Osborne N.R., Davis K.D. (2022). Sex and Gender Differences in Pain. Int. Rev. Neurobiol..

[25] Samulowitz A., Gremyr I., Eriksson E., Hensing G. (2018). “Brave Men” and “Emotional Women”: A Theory-Guided Literature Review on Gender Bias in Health Care and Gendered Norms towards Patients with Chronic Pain. Pain. Res. Manag..

[26] Qu N., Tian H., De Martino E., Zhang B. (2022). Neck Pain: Do We Know Enough About the Sensorimotor Control System?. Front. Comput. Neurosci..

[27] Hesby B.B., Hartvigsen J., Rasmussen H., Kjaer P. (2019). Electronic Measures of Movement Impairment, Repositioning, and Posture in People with and without Neck Pain-a Systematic Review. Syst. Rev..

[28] Meisingset I., Woodhouse A., Stensdotter A.-K., Stavdahl Ø., Lorås H., Gismervik S., Andresen H., Austreim K., Vasseljen O. (2015). Evidence for a General Stiffening Motor Control Pattern in Neck Pain: A Cross Sectional Study. BMC Musculoskelet. Disord..

[29] Kaltenborn F.M. (2010). Movilización Manual de las Articulaciones: Evaluación Articular y Tratamiento Básico. La Columna Vertebral. Volumen II.

[30] Krauss J.R., Evjenth O., Creighton D. (2009). MVT: Manipulación Vertebral Translatoria Para Fisioterapeutas: Mostrando Técnicas de Alta y Baja Velocidad y Corta Amplitud del Concepto Kaltenbron-Evjenth de Terapia Manual.

[31] Rodríguez-Sanz J., Malo-Urriés M., Lucha-López M.O., Pérez-Bellmunt A., Carrasco-Uribarren A., Fanlo-Mazas P., Corral-de-Toro J., Hidalgo-García C. (2021). Effects of the Manual Therapy Approach of Segments C0-1 and C2-3 in the Flexion-Rotation Test in Patients with Chronic Neck Pain: A Randomized Controlled Trial. Int. J. Environ. Res. Public Health.

[32] Kazeminasab S., Nejadghaderi S.A., Amiri P., Pourfathi H., Araj-Khodaei M., Sullman M.J.M., Kolahi A.-A., Safiri S. (2022). Neck Pain: Global Epidemiology, Trends and Risk Factors. BMC Musculoskelet. Disord..

[33] Delorme J., Pennel L., Brousse G., Daulouède J.P., Delile J.M., Lack P., Gérard A., Dematteis M., Kabore J.L., Authier N. (2021). Prevalence and Characteristics of Chronic Pain in Buprenorphine and Methadone-Maintained Patients. Front. Psychiatry.

[34] Stein M.D., Herman D.S., Bailey G.L., Straus J., Anderson B.J., Uebelacker L.A., Weisberg R.B. (2015). Chronic Pain and Depression among Primary Care Patients Treated with Buprenorphine. J. Gen. Intern. Med..

[35] Zahlan G., De Clifford-Faugère G., Nguena Nguefack H.L., Guénette L., Pagé M.G., Blais L., Lacasse A. (2023). Polypharmacy and Excessive Polypharmacy Among Persons Living with Chronic Pain: A Cross-Sectional Study on the Prevalence and Associated Factors. J. Pain. Res..

[36] Dowell D., Ragan K.R., Jones C.M., Baldwin G.T., Chou R. (2022). CDC Clinical Practice Guideline for Prescribing Opioids for Pain—United States, 2022. MMWR. Recomm. Rep. Morb. Mortal. Wkly. Rep. Recomm. Rep..

[37] Hawk C., Whalen W., Farabaugh R.J., Daniels C.J., Minkalis A.L., Taylor D.N., Anderson D., Anderson K., Crivelli L.S., Cark M. (2020). Best Practices for Chiropractic Management of Patients with Chronic Musculoskeletal Pain: A Clinical Practice Guideline. J. Altern. Complement. Med..

[38] Galán-Martín M.A., Montero-Cuadrado F., Lluch-Girbes E., Coca-López M.C., Mayo-Iscar A., Cuesta-Vargas A. (2019). Pain Neuroscience Education and Physical Exercise for Patients with Chronic Spinal Pain in Primary Healthcare: A Randomised Trial Protocol. BMC Musculoskelet. Disord..

[39] Geneen L.J., Moore R.A., Clarke C., Martin D., Colvin L.A., Smith B.H. (2017). Physical Activity and Exercise for Chronic Pain in Adults: An Overview of Cochrane Reviews. Cochrane Database Syst. Rev..

[40] Timmers I., Quaedflieg C.W.E.M., Hsu C., Heathcote L.C., Rovnaghi C.R., Simons L.E. (2019). The Interaction between Stress and Chronic Pain through the Lens of Threat Learning. Neurosci. Biobehav. Rev..

[41] Lynch J., Fox S., D’Alton P., Gaynor K. (2021). A Systematic Review and Meta-Analysis of the Association Between Perceived Injustice and Depression. J. Pain..

[42] Nijs J., Mairesse O., Neu D., Leysen L., Danneels L., Cagnie B., Meeus M., Moens M., Ickmans K., Goubert D. (2018). Sleep Disturbances in Chronic Pain: Neurobiology, Assessment, and Treatment in Physical Therapist Practice. Phys. Ther..

[43] García-Esquinas E., Rodríguez-Sánchez I., Ortolá R., Lopez-Garcia E., Caballero F.F., Rodríguez-Mañas L., Banegas J.R., Rodríguez-Artalejo F. (2019). Gender Differences in Pain Risk in Old Age: Magnitude and Contributors. Mayo Clin. Proc..

[44] Raczkiewicz D., Bejga P., Owoc J., Witczak M., Bojar I. (2020). Gender Gap in Health Condition and Quality of Life at Advanced Age. Ann. Agric. Environ. Med..

